# Note of Subjective Conjunctivitis

**Published:** 1890-12

**Authors:** Jacob Goldberg

**Affiliations:** 145 Cedar Street; Buffalo, N. Y.


### NOTE ON SUBJECTIVE CONJUNCTIVITIS.

By JACOB GOLDBERG, M. D., Buffalo, N. Y.

The condition which I describe under the above title, was brought to my attention by Dr. Bates, of the New York Post-Graduate Medical School, where I attended a course of lectures on the eye and ear, and has been productive of considerable good to me, inasmuch as the text-books do not consider it as here described.

The diagnosis must be made from the statements of the patient, as there are no objective symptoms. He usually states his case about as follows: Asthenopia (headache), slight; photophobia (fear of light), a feeling at times as though there were a particle of sand under the upper or lower lid, which, however, lasts but a moment or two. Upon entering a closed room from the street the eyes water; upon waking in the morning the lids are slightly glued together, as it were, and the use of water is necessary to open them; after reading for one or two hours, or at the theatre from the constant application of the eyes toward the stage, they become red; then the redness quickly fades upon going into the open air and becoming relieved from the strain of constant application; after reading for any length of time, the subject finds that he is almost unable to raise the upper lid.

After receiving the above train of symptoms we find, on examination, everything apparently normal; no redness or swelling of the conjunctiva; no strictures of any kind, or any condition to account for the patient's annoying condition. What are we to do in such cases? In those that I have observed the following plan has been of great benefit: Massage; rubbing the forehead with dry towel will be a service; turkish baths are very good as an aid to relieve any congestion that may exist; a solution of ether, to which may be added lavender or rose water, to be rubbed on the lids for ten or fifteen minutes, will be very serviceable; correct any error of refraction that may exist, although it may only be a half of a diophé of myopia or hypermetropia; order tinted or colored glasses, if photophobia persists; remove any and all irritation, reflex or otherwise; open the bowels by aloes or any cathartic, and finally prescribe constitutional treatment, if indicated.

145 Cedar Street.

